# Effects of Green Tea Extract on Insulin Resistance and Glucagon-Like Peptide 1 in Patients with Type 2 Diabetes and Lipid Abnormalities: A Randomized, Double-Blinded, and Placebo-Controlled Trial

**DOI:** 10.1371/journal.pone.0091163

**Published:** 2014-03-10

**Authors:** Chia-Yu Liu, Chien-Jung Huang, Lin-Huang Huang, I-Ju Chen, Jung-Peng Chiu, Chung-Hua Hsu

**Affiliations:** 1 Institute of Traditional Medicine, School of Medicine, National Yang-Ming University, Taipei, Taiwan; 2 Department of Chinese Medicine, Branch of Linsen and Chinese Medicine, Taipei City Hospital, Taipei, Taiwan; 3 Department of Metabolism, Branch of Linsen and Chinese Medicine, Taipei City Hospital, Taipei, Taiwan; Postgraduate Medical Institute & Hull York Medical School, University of Hull, United Kingdom

## Abstract

The aim of this study is to investigate the effect of green tea extract on patients with type 2 diabetes mellitus and lipid abnormalities on glycemic and lipid profiles, and hormone peptides by a double-blinded, randomized and placebo-controlled clinical trial. This trial enrolled 92 subjects with type 2 diabetes mellitus and lipid abnormalities randomized into 2 arms, each arm comprising 46 participants. Of the participants, 39 in therapeutic arm took 500 mg green tea extract, three times a day, while 38 in control arm took cellulose with the same dose and frequency to complete the 16-week study. Anthropometrics measurements, glycemic and lipid profiles, safety parameters, and obesity-related hormone peptides were analyzed at screening and after 16-week course. Within-group comparisons showed that green tea extract caused a significant decrease in triglyceride and homeostasis model assessment of insulin resistance index after 16 weeks. Green tea extract also increased significantly high density lipoprotein cholesterol. The HOMA-IR index decreased from 5.4±3.9 to 3.5±2.0 in therapeutic arm only. Adiponectin, apolipoprotein A1, and apolipoprotein B100 increased significantly in both arms, but only glucagon-like peptide 1 increased in the therapeutic arm. However, only decreasing trend in triglyceride was found in between-group comparison. Our study suggested that green tea extract significantly improved insulin resistance and increased glucagon-like peptide 1 only in within-group comparison. The potential effects of green tea extract on insulin resistance and glucagon-like peptide 1 warrant further investigation.

**Trial Registration:**

ClinicalTrials.gov NCT01360567

## Introduction

Both type 2 diabetes mellitus (T2DM) and dyslipidemia are individually associated with a cluster of risk factors of atherosclerosis. Lipoprotein abnormalities also increase the thrombotic risk of diabetic patients [1]. Common risk factors for coronary artery disease explain only 25–50% of increased atherosclerotic risk in diabetes mellitus. Other obvious risk factors are hyperglycemia and dyslipidemia. However, hyperglycemia is a very late stage in the sequence of events from insulin resistance to frank diabetes, whereas lipoprotein abnormalities are manifested during the largely asymptomatic diabetic prodrome and contribute to the increased risk of macrovascular disease [2]. Combining medication with lifestyle modification is a logical approach to reduce cardiovascular risk in individuals with dyslipidemia and T2DM [3].

Tea, derived from the plant Camellia sinensis is consumed in the world as green, black or Oolong tea. Among them, green tea has the most significant effects on cardiovascular protection, and the effects of green tea are mainly attributed to its flavonoid-like polyphenols, such as catechins. Catechins concluded mainly epigallocatechin gallate (EGCG), epigallocatechin, epicatechin gallate, and epicatechin, which are the most common green tea extracts (GTE). EGCG is the major catechin in tea and may account for 50–80% of the total catechins in tea [4]. Although several studies implied that EGCG may have the potential to improve the glycemic and lipid profiles in patients with diabetes and dyslipidemia [5]–[8], the effect of GTE on glucose and lipid control was inconsistent, and the underlying mechanism was still unclear. Therefore, this study aims to investigate the effects of decaffeinated GTE on anthropometric measurements, glycemic and lipid profile, as well as hormone levels by a randomized, double-blinded, and placebo-controlled clinical trial.

## Subjects and Methods

This clinical trial was conducted from April 2011 to March 2012 at Taipei City Hospital in Taiwan. Among 236 registered patients with T2DM, whose glycemic hemoglobin higher than 6.5% within 3 months, screened at our outpatient clinic, 102 subjects met the following criteria were enrolled: (1) age between 20 and 65 years, (2) diagnosis of type 2 diabetes for more than one year, (3) body mass index (BMI) ≥ 18 kg/m2 and ≤30 kg/m2, (4) fasting triglyceride ≥ 150 mg/dl or fasting low-density-lipoprotein cholesterol (LDL) ≥100 mg/dl and (5) willing to participate in and fill out questionnaires for this trial. The exclusion criteria include (1) serum alanine transaminase >80 U/L, (2) serum creatinine >1.8 mg/dl, (3) breast feeding or pregnancy, (4) heart failure, acute myocardial infarction, stroke, heavy injury, and (5) any other conditions not suitable for trial as evaluated by the physician. Letters explaining the purpose of the study were sent to all the patients inviting their participation. Finally, with a written informed consent, 102 subjects were enrolled in this study. The protocol was approved by the Human Ethics Committee of Taipei City Hospital. This trial (NCT01360567) was registered with the ClinicalTrials.gov Registry on 20 May 2011 and followed the CONSORT 2010 statements. The protocol for this trial and supporting CONSORT checklist are available as supporting information; see Checklist S1 and Protocol S1.

**Figure 1:**
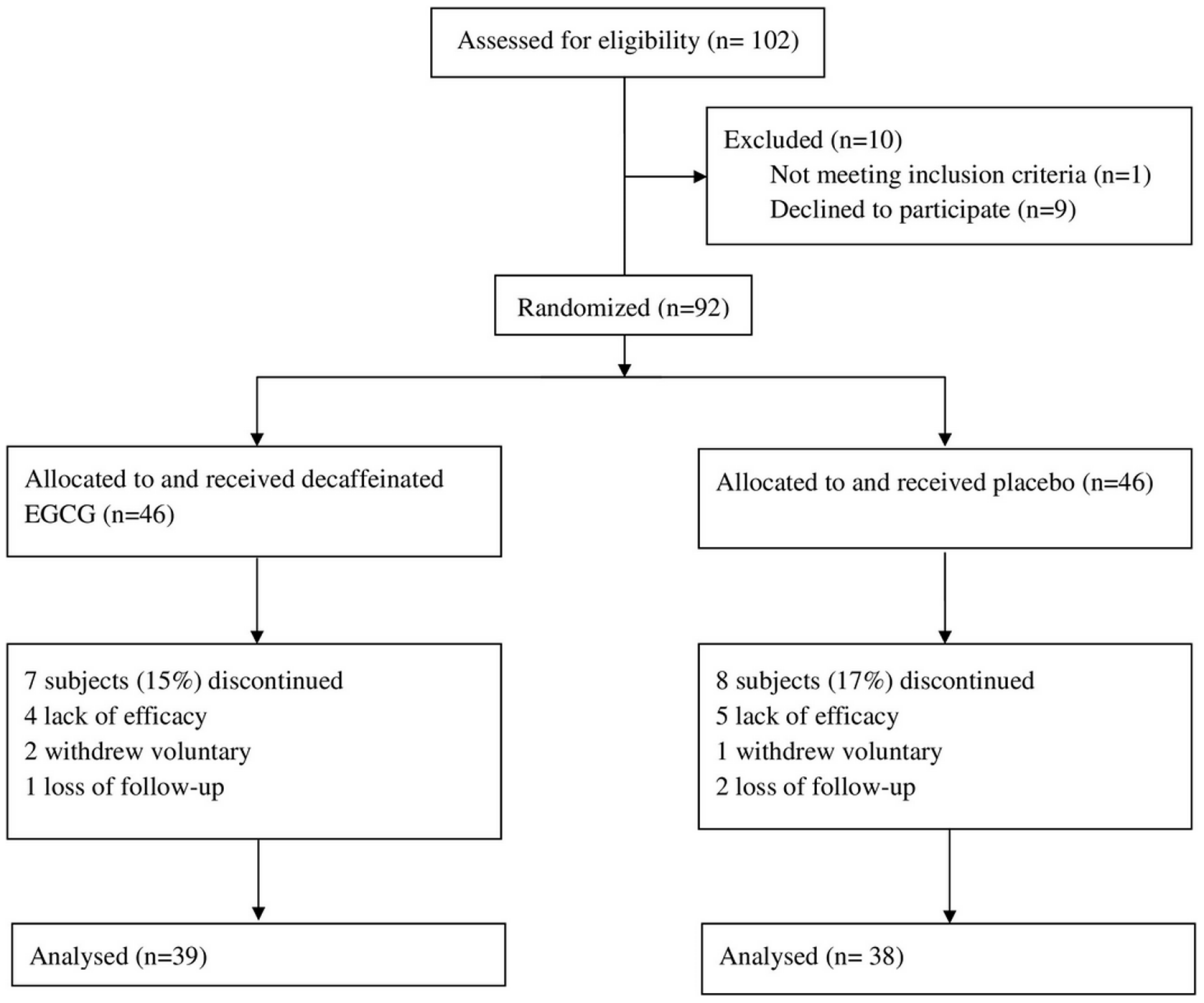
Study Flow Diagram.

Subjects were randomly allocated to receive a decaffeinated GTE EGCG (Group A) or a placebo (cellulose; Group B) for 16 weeks (Figure 1). Both experimental and placebo treatments were contained in the same opaque capsules, which were administered by a blinded research assistant. Subjects were instructed to maintain an isocaloric diet and to continue their previous eating habits during the study period. Every four weeks, subjects returned to our clinics and reported to the study center for adverse events and compliance assessment. Subjects were free to withdraw at any time. Throughout the study period, subjects were directed to continue taking the same dose of any prescribed hypoglycemic agents unless hypoglycemia or severe complications occurred, in which case they were directed to reduce their dose immediately.

### Preparation of samples and treatment

Decaffeinated GTE was obtained from the Tea Research and Extension Station, Taoyuan County, Taiwan. It was extracted from dried leaves of green tea according to pre-set standard procedures. The decaffeinated GTE used in this study was standardized for several tea catechins in addition to EGCG (Table 1). The placebo comprised pure microcrystalline cellulose. Capsules contained either 500 mg decaffeinated GTE extract or cellulose. Subjects were asked to take one capsule 30 minutes after meals three times daily for 16 weeks. Table 1 lists the total daily dose of GTE compounds received by the active group.

**Table 1:** The composition of green tea extracts.

Component	% in weight	Daily dose (in mg)
EGCG (Epigallocatechin gallate)	57.12	856.8
ECG (Epicatechin gallate)	15.74	236.1
EGC (Epigallocatechin)	7.70	115.5
EC (Epicatechin)	4.80	71.9
GCG (Gallocatechin gallate)	4.25	63.7
GC (Gallocatechin)	<0.07	<1.05
Caffeine	<0.07	<1.05
Cellulose	10.3	155.0

### Analysis of obesity-related hormone peptides

The levels of obesity-related hormone peptides, including leptin, insulin, ghrelin, adiponectin, apolipoprotein (apo) A1, apolipoprotein B100, and glucagon-like peptide 1 (GLP-1) (7–36) were measured in the morning after 8–9 hours of fasting. Whole blood sample was drawn and centrifuged at 4°C, with 1 ml aliquot of serum rapidly frozen at −80°C for the subsequent radioimmunoassay concentration analysis. Leptin was detected by the Millipore Human Leptin assay (Millipore, St. Charles, MO, USA) using I125-labled human leptin antiserum with a sensitivity of 0.5 ng/ml for a 100-μL sample. Ghrelin and adiponectin were detected by Millipore Ghrelin RIA Kits (Millipore, St. Charles) and Millipore Adiponectin RIA kits (Millipore, St. Charles) with a sensitivity of 93 pg/ml and 1 ng/ml, respectively. We used the same process as that for leptin detection only with different I125-labled antibodies specific for ghrelin or adiponectin. BioSource INS-IRMA Kits (BioSource Europe S.A., Nivelles, Belgium) were employed to determine the level of insulin in serum as previously reported [9], [10]. The intra- and inter-assay coefficients of variation were 3.1% and 4.9%, respectively. The limit of sensitivity is 0.5 ng/ml. Sampling would be reported if a difference exceeding 10% coefficients of variation was found between duplicated results of the sample. The level of insulin resistance was evaluated by the homeostasis model assessment of insulin resistance index (HOMA-IR), which was calculated with the following model: HOMA-IR  =  insulin [mIU/L] × glucose [mmol/L]/22.5, and values exceeding 2.25 would denote insulin resistance [11]. Circulating level of GLP-1 (7–36) was determined by the Millipore Human glucagon-like peptide-1 RIA kits (Millipore, St. Charles) with a sensitivity of 3 pmol for a 300-μL extracted sample. [12], [13] Apo AI and apo B-100 were detected by immunoturbidimetric assay (K-assay, Kamiya Biomedical Company, Seattle, USA).

### Quality of life

To measure the health-related quality of life (HRQOL) among our subjects, we used the self-administered life-quality questionnaires, world health organization quality of life-BREF (WHOQOL-BREF), Taiwan version which was well validated with consistency coefficients ranging from 0.70 to 0.77 [14]. The WHOQOL-BREF questionnaire evaluated quality of life in physical, psychological, social and environmental domains, with scores ranging from 0 to 100. Higher scores in this questionnaire represent better health condition. Previous study showed that body weight loss could improve HRQOL; hence we use WHOQOL-BREF questionnaire to assess the effects of green tea extracts on quality of life [15].

### Assessment

The single primary outcome was defined as the change of triglyceride (TG) level. The secondary outcomes were evaluated by anthropometric measurements in terms of body weight, height, BMI, waist circumference, and hip circumference. Biochemical characteristics of blood sample including fasting blood sugar, hemoglobin A1c (HbA1c), total cholesterol, low density lipoprotein (LDL)-cholesterol, high density lipoprotein (HDL)-cholesterol levels, high sensitivity C-reactive protein (hsCRP), obesity-related hormone peptides, and HRQOL scores on the WHOQOL-BREF were also assessed. The safety parameters included serum alanine transaminase, creatinine, estimated glomerular filtration rate (eGFR), and adverse event reports. All measurements of biochemical characteristics and obesity-related hormone peptides of blood sample were made at 0800–0900 after an overnight fasting using standardized methods, as detailed in our previous research [16]. All participating physicians received prior training before the study on how to interview the participants and assist them in completing the questionnaires.

### Statistical analysis

The data were analyzed using SPSS software (version 17.0, Chicago, IL.) Independent t-tests were employed to examine the difference in anthropometrics, biochemical characteristics, obesity-related hormone peptides, and life-quality scores between EGCG group and placebo group. Paired t-tests were employed to examine the difference between before and after intervention for 16 weeks in both groups. All p values were two-tailed and α level of significance was set at 0.05. Our own experience suggested a standard deviation (SD) of 3.5% in a previous series of 60 patients with diabetes. It was calculated that to detect a reduction in triglyceride level of 2.4% with a significance level of 0.05 and a power of 0.8, that 64 patients would be required. Allowing for a 10% dropout, 70 patients would be needed in this study.

## Results

Data were analyzed on a per-protocol basis, with subject exclusion occurring before release of the double-blind procedure. A total of 102 subjects were enrolled. Nine subjects did not receive their allocated intervention as they failed to attend study visit because of work commitments or moving out of the area. One subject with poor blood sugar control (fasting plasma glucose values ≥ 500 mg/dL) at baseline was excluded as the primary investigator considered the risk of hyperglycemic hyperosmolar nonketotic coma. In the end, 92 subjects were randomly assigned. Further 7 subjects in the EGCG group and 8 subjects in the placebo group discontinued the intervention. Consequently, the data reported here came from 77 subjects (Fig. 1).

Baseline characteristics for both placebo and EGCG groups are shown in Table 2. No significant differences between any of the group means were detected in demographic, disease duration, glycemic, and lipid parameters (Table 2). To monitor compliance, subjects were required to return all packaging and unused capsules. Each subject received 336 capsules and on average 9.9±13.0 capsules per subject (12.3±15.0 capsules from the EGCG group and 7.5±10.1 capsules from the placebo group) were returned, indicating that more than 95% of doses were taken.

**Table 2:** Demographic data of participants.

	Decaffeinated EGCG (n = 39)	Placebo(cellulose) (n = 38)	P value
Gender (male/female)*	14/25	18/20	0.307
Age (years)	55.0±6.6	53.5±7.0	0.328
Body weight (kg)	66.4±13.2	68.8±13.6	0.442
Body mass index (kg/m^2^)	26.2±4.2	26.4±4.6	0.786
Time since diagnosis of diabetes (years)	5.2±5.9	4.1±4.3	0.339
Family history of diabetes, yes (n)*	27	27	0.861
Fasting blood sugar (mg/dL)	139.2±45.1	152.2±53.5	0.252
Glycemic hemoglobin, HbA1c (%)	7.5±1.6	7.7±1.8	0.591
Low density lipoprotein (mg/dL)	109.7±31.9	115.2±35.4	0.478
Triglyceride (mg/dL)	178.2±105.3	198.3±127.9	0.453
Remnants of capsules, No.	12.3±15.0	7.5±10.1	0.102
(Ratio, remnants/total, %)	(4±4.5)	(2±3)	

* Data analyzed by Chi-square test.

To clarify the effect of concomitant medications on blood pressure, sugar and lipid levels, medications taken by the subjects were reviewed (Table 3). More than half of the participants, 21 subjects (53.8%) in the EGCG group and 23 subjects (60.5%) in the placebo group, took the oral antidiabetes medications. Although the percentage of antidiabetes medication usage was higher in the placebo group, the difference was not statistically significant (p = 0.097). Moreover, 6 subjects (15.4%) in the EGCG group and 4 subjects (10.5%) in the placebo group took antihypertensive medications; while 6 subjects (15.4%) in the EGCG group and 2 subjects (5.3%) in the placebo group took the lipid modifying medications. There was no statistically significant difference in any concomitant medication between the two groups.

**Table 3:** Concomitant medications usage of participants.

Concomitant medication	Decaffeinated EGCG (n = 39)	Placebo(cellulose) (n = 38)	P value
Antidiabetic medications users, %	53.8	60.5	0.097
Sulfonylureas, %	35.9	34.2	0.877
Biguanides, %	43.6	44.7	0.919
Alpha glucosidase inhibitors, %	2.6	5.3	0.541
Thiazolidinediones, %	12.8	5.3	0.249
Dipeptidyl peptidase 4 (DPP-4) inhibitors, %	10.3	5.3	0.414
Meglitinide, %	2.6	0	0.320
Combination, %	35.9	26.3	
Antihypertensives users, %	15.4	10.5	0.787
Angiotensin-converting enzyme inhibitors, %	2.6	2.6	0.985
Angiotensin receptor blockers, %	10.3	7.9	0.719
Calcium channel blockers, %	7.7	2.6	0.317
Combination, %	5.1	2.6	
Lipid modifying agents users, %	15.4	5.3	0.146
HMG CoA reductase inhibitors,%	12.8	5.3	0.249
Fibrates, %	2.6	0	0.320
Combination, %	0	0	

Data analyzed by Chi-square test, except the mean of different medication analyzed by independent t-test.

The 16-week treatment with decaffeinated GTE resulted in decreasing body weight and BMI (p <0.1), compared with baseline measurements, as shown in Table 4a. On the other hands, there is no significant changes in other anthropometric data, including waist circumference, hip circumference, and blood pressure after the 16-week course. Between-group comparison results are listed in Table 5a. There were no statistically significant differences detected for any of the variables assessed after 16 weeks of decaffeinated GTE versus placebo treatment.

**Table 4:** 

Within-group analysis of anthropometrics and biochemical data at baseline and 16 weeks of study						
Variable	Decaffeinated EGCG (n = 39)			Placebo (cellulose) (n = 38)		
	baseline	After 16 weeks	p-value	baseline	After 16 weeks	p-value
Anthropometric data						
Weight, kg	66.4±13.2	66.0±13.3	0.09*	68.8±13.6	68.6±13.7	0.69
Body mass index, kg/m^2^	26.2±4.2	26.0±4.0	0.06*	26.4±4.6	26.4±4.4	0.54
Waist circumference, cm	83.9±9.8	85.2±12.1	0.17	87.8±11.0	87.5±8.4	0.69
Hip circumference, cm	96.4±9.5	96.9±9.3	0.30	99.3±4.0	98.9±10.5	0.30
Waist hip ratio	0.9±0.1	0.9±0.1	0.60	0.9±0.6	0.9±0.5	0.63
Systolic blood pressure, mmHg	133.4±18.6	135.3±18.1	0.48	133.3±16.7	131.6±14.5	0.38
Diastolic blood pressure, mmHg	78.9±10.8	78.1±11.1	0.68	84.5±15.3	80.5±9.1	0.05*
Heart rate, bpm	76.9±13.0	77.6±13.7	0.59	75.9±10.9	76.6±13.1	0.56
Biochemical data						
Alanine transaminase (IU/L)	31.1±17.6	31.8±19.9	0.71	28.1±12.1	28.3±13.1	0.87
Creatinine, mg/dL	0.74±0.17	0.74±0.19	0.68	0.77±0.18	0.77±0.17	1.0
eGFR, %	75.8±13.5	75.2±14.2	0.57	75.6±13.0	75.7±13.3	0.91
Triglyceride, mg/dL	178.2±105.3	159.3±91.6	0.03 **	198.3±127.9	208.2±118.2	0.38
Total cholesterol, mg/dL	195.5±37.4	193.6±40.0	0.67	206.7±47.6	207.4±55.6	0.89
Low density lipoprotein, mg/dL	109.7±31.9	111.8±37.7	0.55	115.2±35.4	111.9±32.4	0.48
High density lipoprotein, mg/dL	49.5±13.6	52.2±15.7	0.04 **	46.7±11.6	46.8±11.4	0.94
Fasting blood sugar, mg/dL	139.2±45.1	148.2±48.1	0.07*	152.2±53.5	151.6±61.7	0.88
Glycemic hemoglobin, HbA1c, %	7.5±1.6	7.5±1.7	0.70	7.7±1.8	7.5±1.7	0.054
HSCRP, mg/L	0.26±0.27	0.36±0.36	0.013*	0.21±0.21	0.28±0.27	0.04*

Within-group analysis of hormone peptides and WHOQOL-BREF at baseline and 16 weeks of study						
Variable	Decaffeinated EGCG (n = 39)			Placebo (cellulose) (n = 38)		
	baseline	After 16 weeks	p-value	baseline	After 16 weeks	p-value
Hormone peptides						
Insulin, IU/L	15.6±10.4	9.3±4.2	0.000**	17.0±14.8	12.3±7.5	0.039*
HOMA-IR index	5.4±3.9	3.5±2.0	0.004**	5.9±4.5	4.7±3.4	0.11
Leptin, ng/mL	7.7±5.4	8.3±5.8	0.46	7.6±7.6	8.1±6.5	0.51
Ghrelin, pg/mL	537.8±255.1	499.7±172.1	0.44	598.4±227.8	440.3±184.3	0.002**
Adiponectin, ug/mL	20.2±5.1	21.7±5.1	0.046**	18.6±2.9	20.3±3.8	0.000**
Apolipoprotein A1, mg/dL	137.9±34.5	191.7±74.5	0.000**	134.5±30.3	165.9±54.5	0.002**
Apolipoprotein B100, mg/dL	95.6±27.6	130.0±62.0	0.001**	99.4±25.9	116.7±40.5	0.006**
Glucagon-like peptide-1 (7–36), pmol/L	1.4±1.2	2.6±1.6	0.001**	1.9±2.3	2.2±1.8	0.60
WHOQOL-BREF						
Physical	62.1±14.1	68.1±12.3	0.001**	66.6±10.8	69.3±14.3	0.08
Psychological	70.9±15.5	72.3±12.1	0.42	74.1±14.5	74.2±12.8	0.95
Social	74.5±13.9	77.4±10.4	0.12	74.8±18.8	76.7±13.6	0.49
Environment	74.6±13.5	79.5±8.9	0.01	75.6±14.1	76.2±11.9	0.75

*p<0.1, **p<0.05

**Table 5:** 

Between-group analysis of anthropometrics and biochemical data at baseline and 16 weeks of study			
	Reduction		
Variable	Decaffeinated EGCG (n = 39)	Placebo (cellulose) (n = 38)	p-value
Anthropometric data			
Weight, kg	−0.7±2.2	−0.2±3.5	0.45
Body mass index, kg/m^2^	−0.2±0.6	−0.1±0.9	0.61
Waist circumference, cm	0.7±4.2	−0.3±4.7	0.35
Hip circumference, cm	0.5±3.2	−0.5±2.6	0.14
Waist hip ratio	−0.0±0.0	0.0±0.0	0.71
Systolic blood pressure, mmHg	1.9±16.9	−1.7±11.7	0.28
Diastolic blood pressure, mmHg	−0.7±11.1	−4.1±12.6	0.23
Heart rate, bpm	0.8±9.1	0.7±7.3	0.96
Biochemical data			
Alanine transaminase (IU/L)	0.7±12.0	0.2±9.1	0.84
Creatinine, mg/dL	0.0±0.1	0.0±0.1	0.76
eGFR, %	−0.7±7.7	0.1±6.9	0.62
Uric acid, mg/dL	0.1±0.8	0.3±0.9	0.34
Triglyceride, mg/dL	−2.1±38.5	16.4±56.6	0.097*
Total cholesterol, mg/dL	−1.9±26.9	0.7±33.6	0.71
Low density lipoprotein, mg/dL	2.1±21.2	−3.3±29.0	0.36
High density of lipoprotein, mg/dL	2.7±7.8	0.1±6.3	0.11
Fasting blood sugar, mg/dL	9.0±30.3	−0.6±25.2	0.13
Glycemic hemoglobin, HbA1c, %	−0.0±5.5	−0.2±0.6	0.24
HSCRP	0.1±0.2	0.1±0.2	0.53

Between-group analysis of hormone peptides and WHOQOL-BREF at baseline and 16 weeks of study			
	Reduction		
Variable	Decaffeinated EGCG (n = 39)	Placebo (cellulose) (n = 38)	p-value
Hormone peptides			
Insulin, IU/L	−6.3±10.0	−4.7±13.5	0.54
HOMA-IR index	−2.0±4.0	−1.3±4.8	0.50
Leptin, ng/mL	0.7±5.6	0.5±4.5	0.88
Ghrelin, pg/mL	−38.1±306.0	−158.1±292.0	0.08*
Adiponectin, ug/mL	1.5±4.4	1.6±2.2	0.24
Apolipoprotein A1, mg/dL	53.9±64.3	31.3±58.7	0.11
Apolipoprotein B100, mg/dL	34.4±57.7	17.3±37.0	0.13
Glucagon-like peptide-1 (7-36), pmol/L	1.1±2.0	0.3±3.0	0.14
WHOQOL-BREF			
Physical	6.0±10.6	2.8±9.5	0.16
Psychological	1.4±10.7	0.1±12.3	0.63
Social	3.0±11.5	1.9±16.8	0.75
Environment	4.9±11.2	0.6±11.1	0.10

*P<0.1

Regarding serum lipid profiles, fasting TG decreased from 178.2±105.3 to 159.3±91.6 with statistical significance in the EGCG group (p =  0.03). However, fasting TG increased from 198.3±127.9 to 208.2±118.2 in the placebo group. In spite of the 2.1±38.5 percent of decrement in EGCG group and the 18.8±58.3 percent of increment in placebo group, decaffeinated EGCG led to a decreasing trend in serum TG (p<0.1). The total cholesterol level decreased slightly from 195.5±37.4 to 193.6±40.0 without statistically significant difference in EGCG group. HDL increased significantly from 49.5±13.6 to 52.2±15.7 in the EGCG group (p = 0.04), but the level after cellulose treatment was almost the same. In the between-group analysis, the percentages of increment in HDL were 6.1±16.0 in the EGCG group and 1.9±17.9 in the placebo group, though without statistical significance. LDL increased from 109.7±31.9 to 111.8±37.7 in the EGCG group but declined from 115.2±35.4 to 111.9±32.4 in the placebo group; and neither difference was statistically significant.

As for blood sugar and insulin resistance, fasting glucose increased from 139.2±45.1 to 148.2±48.1 in the EGCG group, but decreased slightly from 152.2±53.5 to 151.6±61.7 in the placebo group. Neither group showed statistical significance in within-group analysis. With respect to glycohemoglobin, no statistically significant difference existed between the two groups. Interestingly, insulin decreased markedly from 15.6±10.4 to 9.3±4.2 (p = 0.000) in the EGCG group and from 17.0±14.8 to 12.3±7.5 (p = 0.039) in the placebo group. But the difference between groups didn’t was not statistically significant. This study evaluates insulin resistance by HOMA-IR index. The HOMA-IR in the EGCG group decreased from 5.4±3.9 to 3.5±2.0 with statistical significance (p = 0.004), while that in the placebo group decreased from 5.9±4.5 to 4.7±3.4 without statistical significance.

Among the obesity-related hormone peptides, ghrelin in the placebo group decreased from 598.4±227.8 to 440.3±184.3 with statistical significance (p = 0.002), as shown in table 4b. Ghrelin in the EGCG group also decreased after the 16-week course but did not achieve statistical significance. Adiponectin increased markedly in both groups but showed no statistically significant difference between groups. There was also no statistically significant difference in leptin level in the two groups. The hsCRP significantly increased in both groups with difference of 0.1±0.2. Apo AI increased significantly in both groups and the differences were 53.9±64.3 in the EGCG group and 31.3±58.7 in the placebo group. Apo B-100 also increased significantly in both groups and the differences are 34.4±57.7 in the EGCG group and 17.3±37.0 in the placebo group. GLP-1 in EGCG group increased from 1.4±1.2 to 2.6±1.6 with statistical significance (p = 0.001), but the difference in GLP-1 in the placebo group didn’t achieve statistical significance. However, none of the differences between the groups reached statistical significance (table 5b).

### Adverse Effects

In the experimental group, one subject in the experimental group experienced symptoms of epigastric dullness and two developed mild constipation, while one subject in the placebo group had abdominal discomfort. All these symptoms were relieved in the first week after treatment. No major adverse effects of either experimental or placebo group were noted.

## Discussion

The present initial results revealed no statistically significant difference between the EGCG and placebo groups in any of the anthropometric, glycemic, lipid or hormone peptide variables assessed. Despite of adequate sample size after calculation, the metabolic responses to EGCG in those patients with type 2 diabetes were various. Because the small sample size, the difference between the randomized groups was non-significant in our study. But our within-group analysis explored some new findings on GLP-1, insulin resistance, and lipid profile such as triglyceride and HDL, which were less affected by current antidiabetic medications.

The worldwide prevalence of diabetes has continued to increase dramatically instead of the improvement in outcomes for individual patients with diabetes. Lifestyle modification will undoubtedly play a key role in the ultimate solution to the problem of diabetes [17]. The anti-diabetic effect of green tea extracts may raise the potential of green tea to be the lifestyle modification for diabetic prevention. People who drink at least 4 cups of tea per day may have a 16% lower risk of developing type 2 diabetes than non-tea drinkers [18]. However, green tea catechins alone do not positively alter anthropometric measurements. A meta-analysis showed that GTE have a positive effect on weight loss and weight maintenance in obese people (BMI between 25 and 30) [19], [20]. To understand the effect of green tea catechins in anthropometrics of diabetic subjects, and to avoid the potential confounding effect caused by caffeine in green tea, this study used decaffeinated GTE. Although a decreasing trend in body weight and BMI was observed, GTE did not significantly reduce body weight and BMI of diabetic subjects (BMI around 26) in our study. This result is similar to other studies for diabetic rats and diabetic population (BMI around 30) [21], [22]. The difference may be due to the degree of obesity or disease nature, and it merits further investigation.

To our knowledge, this is the first study on the effect of GTE on GLP-1. Our results showed significant within-group changes in GLP-1 level and HOMA-IR index after 16 weeks of treatment only in the GTE group, despite there being no significant change in fasting glucose and HbA1c. Previous research reported a significant interaction between circulating GLP-1, serum HDL, and triglyceride concentrations but not waist circumference, fasting glucose, HbA1c, or presence of diabetes [23]. This result is consistent with the within-group findings in the present study. GLP-1 could lower blood sugar with insulin resistance through up-regulating the pancreatic β-cell to enhance insulin secretion and suppressing glucagon secretion with gastric emptying. Therefore, the incretin therapy has been used in different stages of diabetes in recent years [24]–[26]. Several studies have shown that T2DM patients generally exhibit attenuated GLP-1 secretion [27]–[29]. In our patients, GLP-1 secretion was lower in both groups and enhanced only in GTE group, but whether the levels of GLP-1 secretion were sufficient could not be determined. Thondam et al. reported that metformin increased serum GLP-1 level in T2DM patients [30], and metformin could also enhance GLP-1 secretion in GLP-1 producing cell line [31]. This study has avoided the confounding effects of chronic antidiabetic treatment, because these treatments did not show any significant difference between GTE and placebo groups. Although several studies demonstrated the glucose-lowering effects of GTE, the fasting glucose and HbA1c did not decrease after GTE supplementation in our study. This discrepancy might be due to the more obvious patients with diabetes in our study, whose mean disease duration was around 5 years. The glucose-lowering effects of GTE supplementation are better in patients with diabetes history of less than 5 years than those with a history of more than 5 years. The increased GLP-1, lower insulin, and decreased HOMA-IR were only significant in the population with disease duration of less than 5 years (data was not shown). Future studies with larger numbers of patients will be required to investigate the clinical characteristics of patients with a better GLP-1 response to GTE.

Virtually every lipid and lipoprotein is affected by insulin resistance and diabetes mellitus, but control of hyperglycemia is unlikely to correct existing dyslipidemia. Although plasma glucose control is important in reducing microvascular complications due to diabetes, lipid management is also essential in these patients to decrease the incidence of cardiovascular events [32]. In this study, EGCG significantly reduced fasting triglyceride and increased HDL in within-group analysis and caused a decreasing trend of fasting triglycerides in between-group analysis. Adiponectin, apoA1 and apoB-100 increased significantly in both groups in within-group comparison. Whether adiponectin is positively associated with green tea consumption or not remains controversial [33]–[36]. Green tea extracts also could reduced fat deposit and ameliorated in high fat-fed rats via the adiponectin associated pathway [37]. Since adiponectin plays a role in regulating insulin function and is negatively associated with risk factors for cardiovascular disease, the clinical effects of GTE on adiponectin merits further clarification. ApoAI and apoB-100 are the major apolipoproteins of HDL and LDL, respectively[38]. *In vitro* study revealed that EGCG decreased apoB-100 secretion [39], [40], which is inconsistent with our findings. Increased serum LDL, rather than decreased triglyceride, possibly leads to this discrepancy. However, the increase in apoAI after EGCG supplementation is compatible with that in HDL. Current guidelines recommend statin therapy and lifestyle modification as primary intervention for reducing cardiovascular risk in patients with T2DM. However, even with intensive lowering of LDL cholesterol, patients remain at high residual risk because of low HDL cholesterol and/or elevated triglycerides, justifying the need for additional therapy specifically aimed at the management of these abnormalities [3]. EGCG may be the logical dietary supplement for combination with a statin in this setting. Large prospective studies are needed to evaluate the clinical benefits and tolerability of these combinations.

Although the literature reported several suspected green tea-related hepatic reactions [41], the hepatotoxicity is probably due to metabolism or concomitant medications of a particular patient. Neither impaired liver/renal function nor major adverse effects were seen in our study. As for the limitation of this study, the relatively small sample size and relatively large variance of the metabolic measurements made the outcome non-significant. Besides, the compliance was monitored only by counting the returned packages and unused capsules. This study didn’t measure the participants' serum level of EGCG to confirm the bioavailability and the effects on metabolism. Further research on the bioavailability and pharmacokinetics of EGCG in human studies is needed.

In conclusion, the present study showed no statistically significant difference between decaffeinated GTE and placebo in anthropometrics, glycemic and lipid profiles, as well as obesity-related hormone peptides after 16 weeks of treatment. Daily taking decaffeinated GTE with dose of 856 mg EGCG for 16 weeks is safe and free of severe adverse effects. The metabolic effects on GLP-1 and insulin response of decaffeinated GTE in humans warrant continued investigation.

## Supporting Information

Checklist S1 (DOC)

Protocol S1 (DOCX)
