# Supplementary material for: Effects of Green Tea Extract on Insulin Resistance and Glucagon-Like Peptide 1 in Patients with Type 2 Diabetes and Lipid Abnormalities: A Randomized, Double-Blinded, and Placebo-Controlled Trial
Source: PLoS One. 2014 Mar 10;9(3):e91163. doi: 10.1371/journal.pone.0091163 (PMC3948786; doi:10.1371/journal.pone.0091163)
Supplement: Protocol S1 — (DOCX) [file pone.0091163.s002.docx]

| **4. Study protocol** |
| --- |
| 1. **Study population**   The study participants will be enrolled from the outpatient clinics of internal medicine department, family medicine department and Chinese medicine department of Linsen and Chinese medicine branch of Taipei City hospital.   1. **Study period**   2011/01/01 to 2012/12/31   1. **Study group**   2 groups   1. **Study duration**   16 weeks   1. **Study design**   Double blinded, randomized, and placebo-controlled trial   1. **Randomization**   The randomization sequence will be generated by a table of random numbers from the SPSS software. The allocator will have to call a central number to receive the next allocation sequence once a patient had been enrolled in the trial.   1. **Primary endpoint** 2. The percentage of change between baseline and 16 weeks in fasting triglyceride, total cholesterol, HDL, LDL. 3. Anthropometric measurements in terms of body weight, height, BMI, waist circumference, and hip circumference.   **8. Secondary endpoint**   1. The percentage of change between baseline and 16 weeks in fasting sugar, HbA1C, obesity related hormone peptides, such as leptin、adiponectin、ghrelin、fasting insulin resistance、APOB、APOA1, high sensitivity C-reactive protein (hsCRP) 2. HRQOL scores on the WHOQOL-BREF   Assessment method:  All measurements of biochemical characteristics and obesity-related hormone peptides of blood sample will be made at 0800-0900 after an overnight fasting using standardized methods. All measurements will do after an overnight fast using standardized method and will be performed in the beginning of the study and after 16 weeks of treatment. Height is measured with a wall-mounted stadiometer to the nearest 0.1 cm, weight is measured on a calibrated balance beam scale to the nearest 0.1 kg, and BMI is calculated according to the formula: BMI = body weight (BW) / height (kg/m^2^). We simultaneously collected the demographic data and fasting laboratory data such as blood sugar, creatinine, aminotransferases aspartate, aminotransferases alanine, uric acid, and plasma lipoproteins (triglyceride, cholesterol, HDL-cholesterol (HDL) and LDL-cholesterol (LDL)). Homeostasis model assessment for insulin resistance (HOMA-IR)【fasting glucose (mmol/l) × fasting insulin (UI/l)/22.5】 is used as insulin resistance measurement (25-26). All measurements will make at 0800-0900 after an overnight fast using standardized methods. A sample of whole blood was drawn and centrifuged at 4°C, and a 1-ml aliquot of serum was rapidly frozen (-80°C) for subsequent hormone analysis. The plasma adiponectin concentration will be measured by a radioimmunoassay kit (Linco Research, Inc., St. Charles, MO, USA). This kit employs the double-antibody/ polyethylene glycol technique using ^125^I-lableled adiponectin and a multispecies adiponectin rabbit antiserium. Plasma insulin levels will be measured using a commercially available radioimmunoassay (Linco Research, Inc., St. Charles, MO, USA). Plasma ghrelin levels will be measured with a commercially available radioimmunoassay (Linco Research, Inc., St. Charles, MO, USA), using ^125^I-lableled ghrelin as a tracer. The detection limitation for the assay will be 10 ng/ml.  **9. Safety evaluation**  （1）adverse event  （2）vital signs  （3）Physical examination  　 （4）laboratory evaluation including liver function and renal function  **10. Inclusion criteria**   1. Age between 20 and 65 years 2. Diagnosis of type 2 diabetes for more than one year 3. Body mass index (BMI) ≥ 18 kg/m2 and ≤ 30 kg/m2 4. Fasting triglyceride ≥ 150 mg/dl or fasting low-density-lipoprotein cholesterol (LDL) ≥ 100mg/dl 5. Willing to participate in and fill out questionnaires for this trial   **11. Exclusion criteria**   1. Serum alanine transaminase > 80 U/L 2. Serum creatinine > 1.8 mg/dl 3. Breast feeding or pregnancy 4. Heart failure, acute myocardial infarction, stroke, heavy injury 5. Any other conditions not suitable for trial as evaluated by the physician   **12. study protocol:**  All participants met the criteria will be asked to sign the informed consent form after letters explaining the purpose of the study sent to all the patients inviting their participation. Participants will be randomly allocated to receive a decaffeinated GTE EGCG (Group A) or a placebo (cellulose; Group B) for 16 weeks. Both experimental and placebo treatments were contained in the same opaque capsules, which will be administered by a blinded research assistant. Participants will be instructed to maintain an isocaloric diet and to continue their previous eating habits during the study period. Every four weeks, Participants will return to our clinics and report to the study center for adverse events and compliance assessment. Participants will be free to withdraw at any time. Throughout the study period, participants will be directed to continue taking the same dose of any prescribed hypoglycemic agents unless hypoglycemia or severe complications occurred, in which case they will be directed to reduce their dose immediately.  **13. Adverse event report**  We will assess any adverse event of the participants during the study and will discuss the necessary to drop out.  **14. Drop out/ withdraw criteria**   - 1. drug compliance < 60% or no willing to return during the study   2. pregnancy occurred during the study   3. major illness or hospitalization occurred during the study   4. the dose of antidiabetic or antihyperlipidemia medication changed during the study   5. not suitable to participant after doctor assessed during the study   6. lost of follow up or death   **15. Statistical analysis**   1. The data will be analyzed using SPSS software (version 17.0, Chicago, IL.) 2. Independent t-tests will be employed to examine the difference in anthropometrics, biochemical characteristics, obesity-related hormone peptides, and life-quality scores between EGCG group and placebo group. 3. Paired t-tests will be employed to examine the difference between before and after intervention for 16 weeks in both groups. 4. All p values will be two-tailed and α level of significance will be set at 0.05. 5. This study population will be “Intent to treat population” for analysis of therapeutic efficacy 6. The definition of ITT （Intent to treat population）is that participants take the study medication once.   **16. Preparation of samples and placebo**  Decaffeinated GTE was obtained by Dr. Chen from the Tea Research and Extension Station, Taoyuan County, Taiwan. It was extracted from dried leaves of green tea according to pre-set standard procedures. The decaffeinated GTE used in this study was standardized for several tea catechins in addition to EGCG. The placebo comprised pure microcrystalline cellulose. Capsules contained either 500 mg decaffeinated GTE extract or cellulose. This sample contains 57.12% EGCG (80.83% Total catechines.  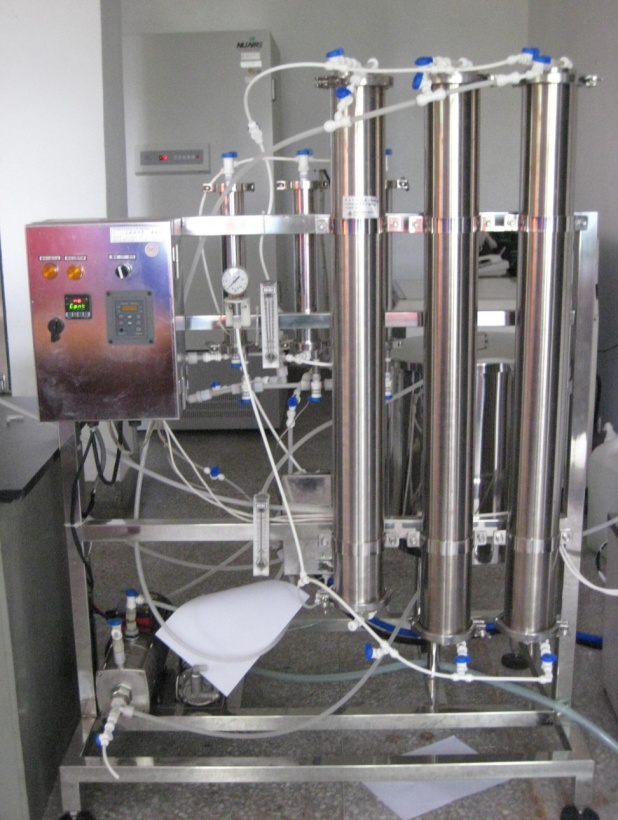  （1）The machine was used to extract decaffeinated GTE.  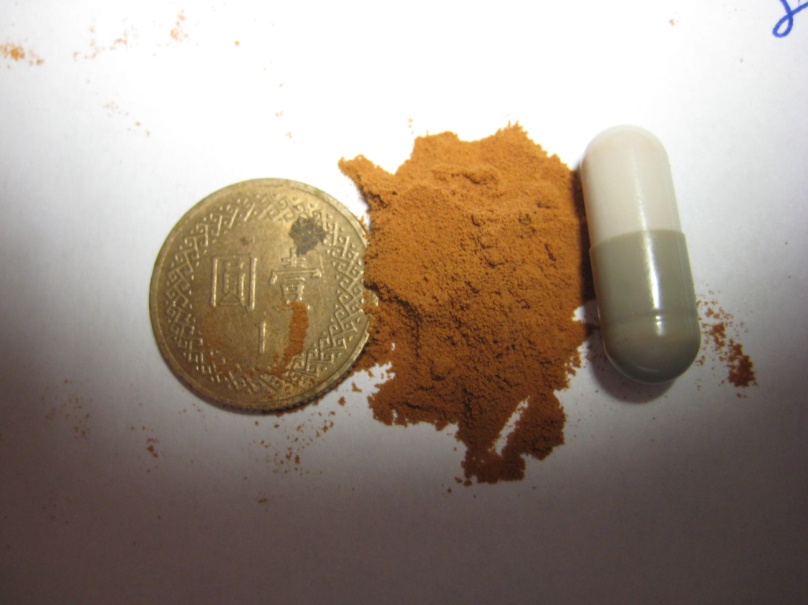  （2）Placebo (B group) was made by cellulose. |
